# Multimodal Multidirectional Piezoelectric Vibration Energy Harvester by U-Shaped Structure with Cross-Connected Beams

**DOI:** 10.3390/mi13030396

**Published:** 2022-02-28

**Authors:** Hongbo Qin, Shuting Mo, Xin Jiang, Siyao Shang, Peng Wang, Yan Liu

**Affiliations:** 1 Key Laboratory of Electronic Equipment Structure Design, Ministry of Education, Xidian University, Xi’an 710071, China; qhb0920qhb@xidian.edu.cn (H.Q.); stmo@stu.xidian.edu.cn (S.M.); 21041212042@stu.xidian.edu.cn (X.J.); syshang@stu.xidian.edu.cn (S.S.); 2 Shaanxi Key Laboratory of Industrial Automation, School of Mechanical Engineering, Shaanxi University of Technology, Hanzhong 723001, China; wangpeng@snut.edu.cn

**Keywords:** vibration energy, piezoelectric harvester, U-shaped structure, cross-connected beams

## Abstract

This paper proposes a multidirectional piezoelectric vibration energy harvester based on an improved U-shaped structure with cross-connected beams. Benefitting from the bi-directional capacity of U-shaped beam and additional bending mode induced by cross-connected configuration, the proposed structure can well capture the vibrations in 3D space at the frequencies lower than 15 Hz. These features are further validated by finite element analyses and theorical formulas. The prototype is fabricated and the experiments under different conditions are carried out. The results show that the proposed harvester can generate favorable voltage and power under multidirectional vibrations at a low operating frequency. Practical applications of charging capacitors and powering a wireless sensor node demonstrate the feasibility of this energy harvester in supplying power for engineering devices.

## 1. Introduction

The large-scale use of fossil fuels is causing serious impacts on the global climate in the past few decades. Lowering carbon emission has become a critical task for the global to pursue carbon neutrality. Recently, the issue of harvesting clean and renewable energy from ambient environment has been promoted to provide a possible alternative to the conventional solid-state batteries in the booming IoT world. Harvesting energy from ambient vibrations, such as winding, human motion, vehicles, machines and buildings, etc., have been reorganized as a feasible solution to supply green energy to wireless sensor nodes [1,2,3,4,5,6], which can be implemented by using electromagnetic [7,8], electrostatic [9] and piezoelectric [10,11] transduction mechanisms to convert mechanical energies into electricity. Among them, piezoelectric vibration energy harvesters (PEVHs) have been highlighted because of its high-power density, ease of implementation and miniaturization [5,12,13]. Typically, a PEVH can be constructed by a proof mass to capture the vibration, a piezoelectric plate to convert the energy and a substrate to support the mass and plate [14]. Though changing the configuration of mass and substrate can adapt PEVH’s characteristics with various energy sources, a PVEH for capturing multidirectional, inconstant environmental vibrations is still an investable research for reusing wasted ambient environment energy [15].

Lowering the resonant frequency and expanding efficient bandwidth are two feasible approaches for PVEH to optimize its dynamic properties. The low resonant frequency can be realized by decreasing the structural stiffness or increasing the weight of proof mass, which is easy but may reduce the long-term reliability of devices [16,17,18]. A more viable strategy is frequency up-conversion mechanism, in which the targeted vibration is captured by a low-frequency structure and transmitted to a high-frequency oscillator to trigger its free oscillation [19,20]. Consequently, this scheme can well capture the low-frequency vibrations and then realize a high-frequency, efficient mechanical-electrical conversion. In terms of broadband harvesting, multimodal approach and structural nonlinearity are often utilized [21,22]. The former can produce several resonant peaks in a certain frequency range with the helps from segmented beam-mass and multi-unit configurations [23,24]. Then, the nonlinearity, induced by internal force or large deformation, can increase the width of each resonant peak, giving the PVEH a better coverage to the spectrum of volatile vibration [25,26].

For multidirectional harvesting, a direct method is arranging harvesting units for every interesting direction, such as the dandelion-like structure with 13 piezoelectric cantilevers [27], and the multi-beam structure with coupling magnets [28]. These schemes can effectively capture the input vibrations, but their large, bulky structures will inevitably lead to a poor volume efficiency. Thus, many efforts are devoted to the development of single-unit device with the ability of capturing vibration in multiple directions. For instance, the structures combining pendulum and cantilever or U-shaped beam have been utilized to compose multidirectional PVEHs [29,30,31], but their heavy ball, large range for ball swing and fixed installation direction may hinder their extensive application. A more compact scheme based on twist piezoelectric beam gives the PVEH a capacity of generating a substantial amount of power under the excitation from any direction in the plane in parallel with the beam cross-section [32]. However, its first two resonant frequencies are far from each other and cannot well match the frequency spectrum of ambient vibrations. Consequently, there are still great demands on developing multimodal, multidirectional PVEHs that can efficiently harvest energy from excitations in real-world environment.

This paper proposed a PVEH based on an improved U-shaped structure for effectively harvesting energy from the multidirectional, inconstant vibrations in daily life. Benefitting from cross-connected beams in the legs of U-shaped structure, this device can sense the stimulations in all the planes that parallel and perpendicular with U character. The adhered piezoelectric plates can convert stress energy in cross-coupled legs and horizontal beam into electric energy based on the direct piezoelectric effect. By optimizing the structural dimension, the proposed device can exhibit multi-modal characteristics in the frequency of 2–15 Hz. First, a simulation model of proposed PVEH is established for finite element analysis (FEA), and its static and dynamic characteristics are evaluated. A simplified mode for formulizing the structural resonant frequency is constructed. Then, an experimental prototype is prepared and tested by a tailored setup to verify the harvesting performances and prove the design and analysis results. Necessary discussions and practical applications are conducted. Finally, main findings and conclusions are summarized.

## 2. Design and Simulation

### 2.1. Design and Working Principle

**Figure 1:**
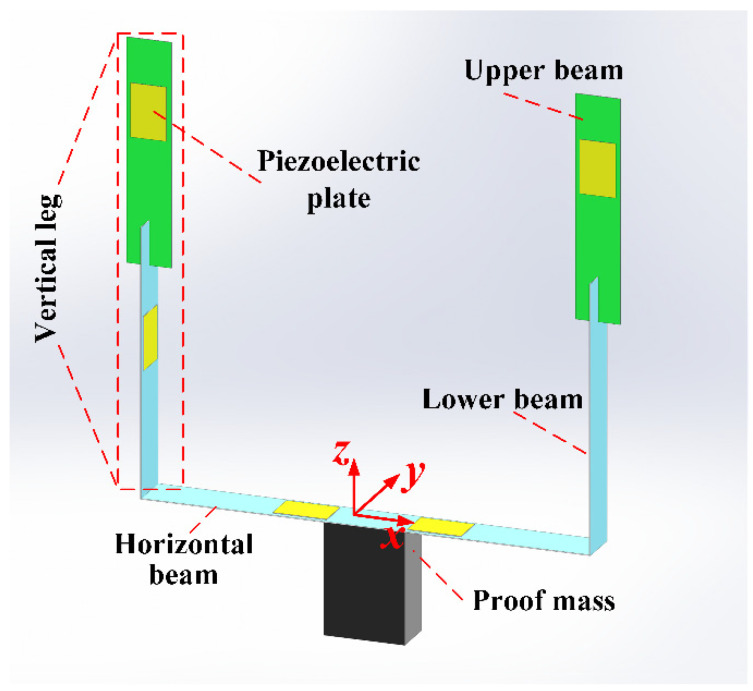
The proposed U-shaped structure with cross-connected beams.

Figure 1 illustrates the proposed multidirectional PVEH, which is mainly composed of the vertical legs with cross-connected beams (viz. upper beams and lower beams), a horizontal beam between the legs, a proof mass on the center of horizontal beam, and six piezoelectric plates uniformly adhered onto the horizontal and cross-connected beams. The vertical legs and horizontal beam form a U-shaped configuration and play the role of responding to 3D space vibrations. For convenience, a Cartesian coordinate system is also defined in Figure 1, whose axes are respectively parallel to the orthogonally distributed normal directions of horizontal beam and two cross-connected beams. The proposed structure can be regarded as a conventional U-shaped beam plus two cross-connected upper beams. The conventional U-shaped structure produces two operating modes in vertical and horizontal directions (namely *x*- and *z*-axis directions). The two upper beams can generate a new bending deformation along *y*-axis, bringing the device more superior performances in harvesting 3D space vibrations. It can be expected that regardless of the direction of loaded excitation, the flexural deformation of corresponding beams can guarantee the activation of their piezoelectric transducers for energy conversion.

### 2.2. Analysis for Proof-of-Concept

For proof-of-concept, a finite element model of the proposed PVEH is built. The whole U-shaped substrate is made of brass, the proof mass is chosen as steel, and the material of piezoelectric plates is PZT-5A. The mass weight is 30 g, and the dimensions of other parts are listed in Table 1. The connected region (about 5 mm) in each vertical leg is not included in the valid length of cross-connected beams. The material parameters in FEA are given as (Elastic modulus, Destiny and Poisson’s ratio): 90 GPa, 8540 kg/m^3^ and 0.38 for brass, 60 GPa, 7750 kg/m^3^, and 0.36 for PZT-5A.

**Figure 2:**
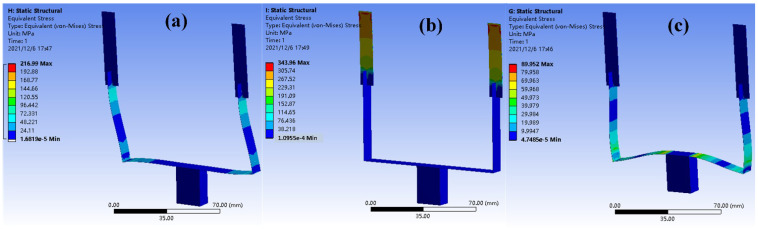
The stress distribution in the proposed U-shaped structure when stimulated by (**a**) *x*-axis, (**b**) *y*-axis, and (**c**) *z*-axis accelerations.

Static analysis is conducted by loading a 1 g (1 g = 10 m/s^2^) acceleration to the proposed model. Figure 2a shows the distribution of equivalent stress under *x*-axis acceleration, in which the corresponding stress mainly concentrates in the lower beam of vertical leg; the *y*-axis acceleration can produce a stress concentration near the fixed end of upper beam, as shown in Figure 2b. Then, the maximum stress under a *z*-axis acceleration symmetrically distributes near both sides of proof mass. Thus, these three stress concentrated regions are chosen to paste piezoelectric ceramics to maximize the sensed mechanical energy.

**Figure 3:**
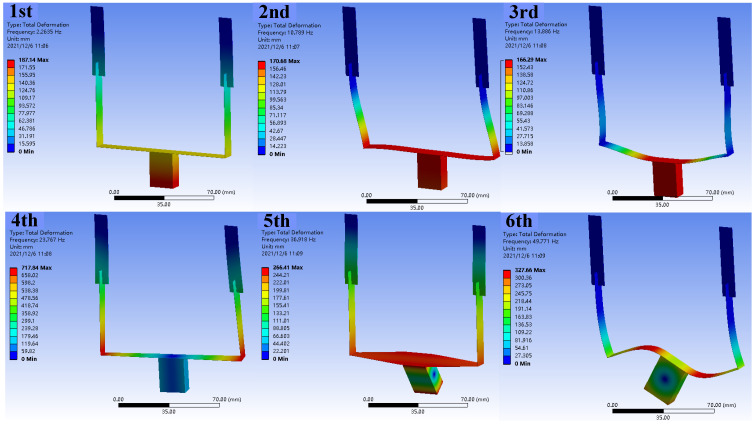
The first six modes of proposed U-shaped structure.

Figure 3 illustrates the first six-order modes of proposed U-shaped structure, and the resonance happens at the frequencies of 2.26 Hz, 10.79 Hz, 13.89 Hz, 23.77 Hz, 36.92 Hz, 49.77 Hz, respectively. The first-order mode is dominated by the flexural deformation of upper beams, corresponding to the additional working direction of U-shaped beam. The second-order and third-order modes are the horizontal and vertical modes that similar with conventional U beam, while the upper beam does not deform. The fourth-order mode shows a torsional deformation of upper beams, and the fifth- and sixth-order modes are dominated by the high-order modes of conventional U beam. It can be seen that the first three modes are all in the form of flexural deformation and constitute the primary working forms of propose PVEH. Meanwhile, the last three torsional deformations are not very suitable for energy harvesting and will not be concerned in the following sections.

**Figure 4:**
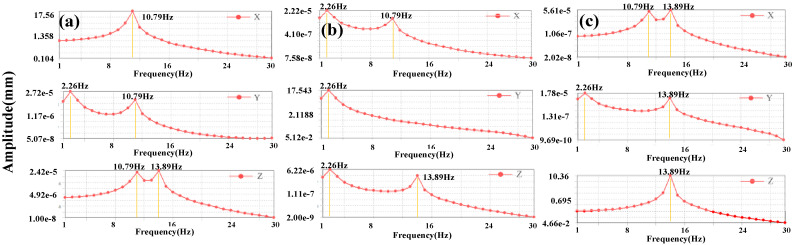
The deformations from harmonic response analysis when the U-shaped structure excited by (**a**) *x*-axis, (**b**) *y*-axis, and (**c**) *z*-axis accelerations.

Further, harmonic response analysis is conducted to verify the effect from external excitation on the performance of PVEH. The 1 g sinusoidal acceleration with frequencies ranging from 0–30 Hz are applied in different directions. As shown in Figure 4a, the *x*-axis acceleration triggers a distinct deformation in the direction of *x*-axis at the frequency of 10.79 Hz, and the deformations in other two directions are much lower. This further indicates that the second-order mode of U-shaped structure effectively undertake the task of collecting vibrations in *x*-axis direction. Similarly, the distinct deformation triggered by *y*-axis acceleration is in the direction of *y*-axis at the frequency of 2.26 Hz, dominated by the first-order of structure. Also, the third-order mode of 13.89 Hz exhibits a better efficiency when harvesting the *z*-axis acceleration.

Concerning the FEA results, the first three modes, namely operating modes of proposed PVEH, correspond to the bending deformation of upper beams, first horizontal mode and vertical mode of conventional U beam, respectively. Thus, the improved U-shaped structure can be divided into two parts to derive the formulas for its working frequencies. Generally, the working frequency can be expressed as

(1) $$\omega_{i}=\sqrt{\frac{K_{i}}{M_{i}}}$$

where *K_i_*, *M_i_* are the equivalent stiffness and mass of whole structure.

For the first mode, equivalent stiffness *K_iu_* is determined by the bending of two upper beams and *M_iu_* is dominated by the mass distribution of lower parts, including proof mass, lower beams, and horizontal beam. Thus,

(2) $$M_{iu}=\frac{33}{140}mL+M_{t}$$

(3) $$K_{iu}=\frac{6EI}{L^{3}}$$

where *L* is the length of upper beam, *m* is the mass per unit length of beams, *M_t_* is mass of proof mass. *EI* is the flexural rigidity of upper beam, which can be obtained by analyzing the feature of piezoelectric cantilever.

The next two modes are similar with the conventional U-shaped beam. So, the equivalent stiffness and mass for horizontal (indicated by the subscript *h*) and vertical (indicated by the subscript *v*) modes are

(4) $$K_{h}=\frac{\frac{l_{1}l_{2}{}^{2}}{EI_{1}}+\frac{l_{2}{}^{3}}{6EI_{2}}}{\frac{l_{1}{}^{3}}{6EI_{1}}\left(\frac{l_{1}l_{2}{}^{2}}{EI_{1}}+\frac{l_{2}{}^{3}}{6EI_{2}}\right)-\frac{1}{8}{\left(\frac{l_{1}{}^{2}l_{2}}{EI_{1}}\right)}^{2}}$$

(5) $$K_{v}=\frac{\frac{l_{1}{}^{2}}{2EI_{1}}+\frac{l_{1}l_{2}}{EI_{2}}}{\left(\frac{l_{1}l_{2}{}^{2}}{8EI_{1}}+\frac{l_{2}{}^{3}}{48EI_{2}}\right)\times \left(\frac{l_{1}{}^{2}}{2EI_{1}}+\frac{l_{1}l_{2}}{EI_{2}}\right)-\left(\frac{l_{1}l_{2}}{2EI_{1}}+\frac{l_{2}{}^{2}}{8EI_{2}}\right)\times \left(\frac{l_{1}{}^{2}l_{2}}{8EI_{1}}+\frac{l_{1}l_{2}{}^{2}}{8EI_{2}}\right)-\frac{3l_{1}{}^{2}l_{2}{}^{2}}{64EI_{1}EI_{2}}}$$

(6) $$M_{v}=M_{t}+\alpha_{v}M_{1}+\beta_{v}M_{2}$$

(7) $$M_{h}=M_{t}+M_{2}+\alpha_{h}M_{1}+\beta_{h}M_{2}$$

where *EI*_1_ and *EI*_2_ are the flexural rigidity of lower and horizontal beams, respectively. *l*_1_ and *l*_2_ are their lengths. *M*_1_ and *M*_2_ are mass of lower and horizontal beams. *α_i_* and *β_i_* are the coefficients determined by structural configuration. More information can be found in [33].

## 3. Prototype and Experimental Setup

**Figure 5:**
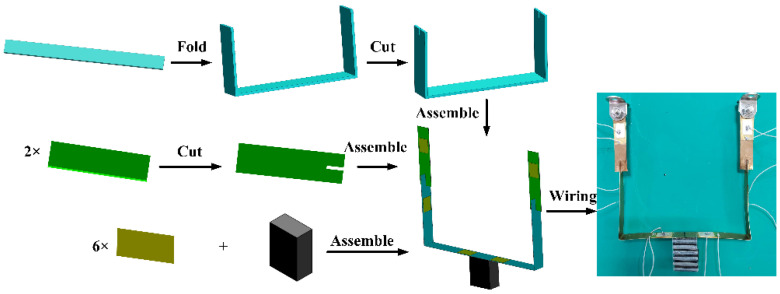
Manufacturing procedure for the proposed PVEH.

An experimental prototype of proposed PVEH is manufactured, following the procedure shown in Figure 5. The whole U-shaped structure can be divided into four parts: the horizontal beam and two lower beams in vertical legs, the upper beams, an iron block as proof mass, and six PZT-5A plates. The PZT-5A plate contains an upper silver electrode, a bottom brass substrate and a piece of polarized piezoceramics. First, a long brass strip was folded to form a conventional U-shaped beam, which corresponded to the part of horizontal beam and two lower beams of PVEH. Two openings were cut by a scissor at each end. Then, two short brass strips for upper beams were prepared, and opening was also cut at one end of each strip. Next, the short brass strips were assembled to the existing U-shaped beam by perpendicularly sticking the openings together, forming a cross connection between the upper and lower beams. Finally, the proof mass and six PZT-5A plates were adhered onto the U-shaped structure in the determined region by an insulating glue.

**Figure 6:**
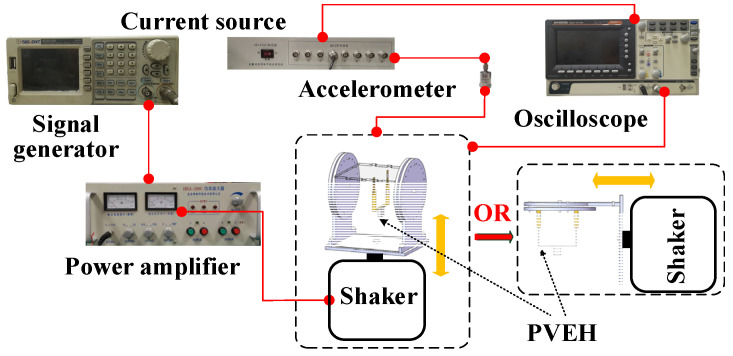
Experimental setup for characterization.

An experimental setup for characterization is shown in Figure 6. The signal generator (SDG1020, SIGLENT, Shenzhen, China) provides a sinusoidal voltage to the power amplifier (HEA-200C, Foneng, Nanjing, China), which is used to excite the shaker (HEV-200, Foneng, Nanjing, China). An oscilloscope (GDS-1072B, GWINSTEK, Suzhou, China) is utilized to measure the voltage generated by PVEH and monitor the acceleration obtained from accelerometer (CA-YD-180, BDHSD, Qinhuangdao, China). The current source (SD14T03, BDHSD, Qinhuangdao, China) is used to power the accelerometer and processing its output signal.

**Figure 7:**
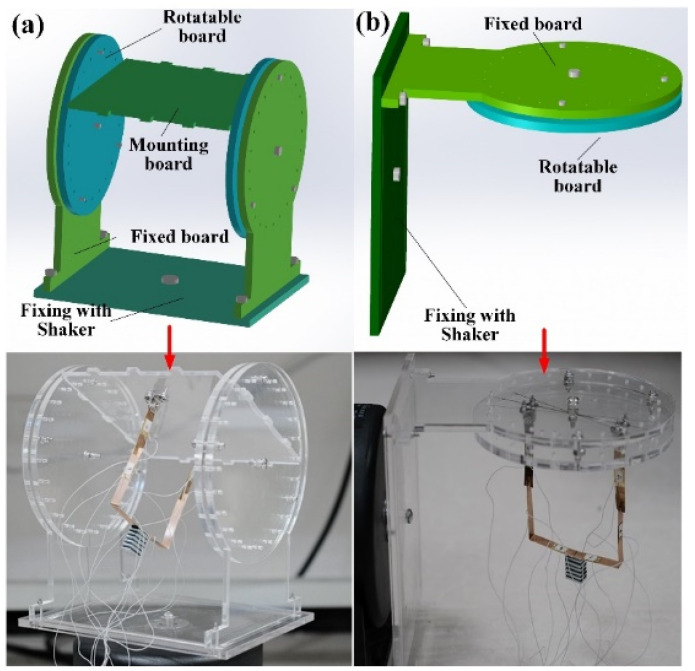
Tailored clampers for (**a**) vertical and (**b**) horizontal vibrations.

To verify the multidirectional harvesting capacity, two acrylic clampers are designed to adjust the angles between stimulating direction and orientation of prototype. The clamper in Figure 7a is used to rotate the PVEH around *y*-axis under the vertical vibrations, adjusting the angle between stimulating direction and *z*-axis of PVEH (defined as α). α = 0° and 90° indicate the stimulating direction is aligned with *z*-axis and *x*-axis of PVEH. The clamper in Figure 7b is used when the vibration is horizontally loaded. The PVEH is rotated around *z*-axis to adjust the angle between stimulating direction and *y*-axis of PVEH (defined as β). β = 0° and 90° indicate the stimulating direction is aligned with *x*-axis and *y*-axis, respectively.

## 4. Results and Discussions

With the help of experimental setup and rotatable champers, the prototype is successively stimulated by the vertical and horizontal vibrations with different angles. Due to the symmetry of U-shaped structure, the PVEH will generate similar response when the angle is beyond 90°, so the experiments are conducted in the range of 0° to 90°. In the experiments, the prototypes are firstly triggered by the loaded vibrations to obtain the curves of open-circuit voltage versus excitation frequency under different α or β. Under the vertical vibration, the third-order mode of U-shaped structure will be triggered at the beginning, and the lower beam will be bent when α increases. The horizontal vibrations mainly triggered the first- and second-order modes of U-shaped structure when β varies. Then, the power tests are conducted by recording the voltage over the loaded resistors when the devices are stimulated at their optimized frequencies. Last, some applications are demonstrated by charging capacitors and powering a wireless sensor node. All the experiments are conducted at room temperature about 20 °C and a humidity of 40% RH.

### 4.1. Experimental Results

**Figure 8:**
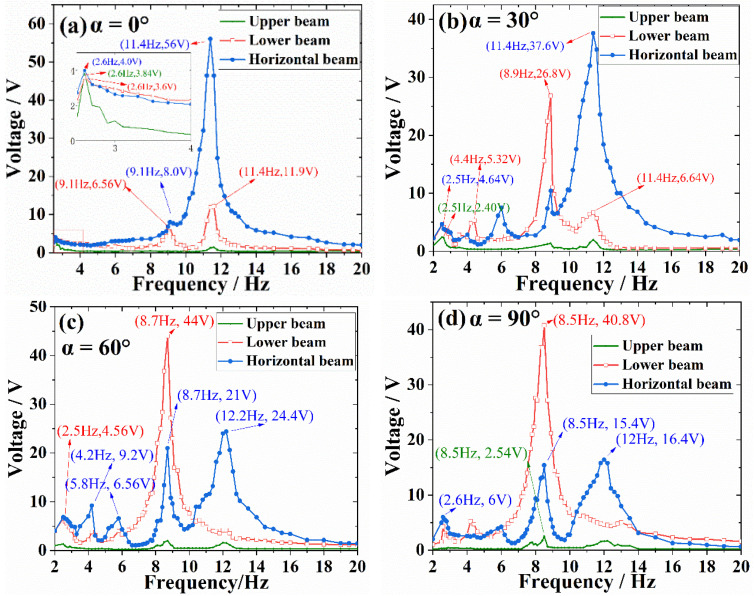
Open-circuit voltage versus frequency under vertical excitation with different α.

Figure 8 shows the open-circuit voltage of PVEH with α varying from 0° to 90° when the 1 g acceleration is vertically loaded. When α = 0°, three peaks are generated at the frequency of 2.6 Hz, 9.1 Hz, and 11.4 Hz, which is similar to the simulation results of modal and harmonic response analysis. At 2.6 Hz, the small oscillation of PVEH bends the upper beams, and the lower, and horizontal beams are also deformed by the effect of gravity, whose output voltages are all around 4 V. At 9.1 Hz, the structure exhibits a horizontal mode with voltages of 6.56 V for lower beams and 8.00 V for horizontal beam. At 11.4 Hz, the structure exhibits a vertical mode and the horizontal beam is strongly deformed with an output voltage of 56 V. When α = 30°, the horizontal mode at second peak becomes more intense, inducing a higher voltage of 26.8 V in the lower beam. Meantime, the output voltage of horizontal beam decreases to 37.6 V at 11.4 Hz, due to the reduction of effective acceleration component. Figure 8c illustrated the circumstance of α = 60°, in which the voltage of lower beam reaches the value of 44 V, and the voltage of horizontal beam further decreases to 24.4 V. When α = 90°, the excitation direction is perpendicular to upper beam plane, and the output voltage frequency response is shown in Figure 8d. Except of the three resonant peaks corresponding to the results of mode analysis, there are additional mini peaks near 4.4 Hz and 5.8 Hz in the curves of upper and horizontal beams when α = 30°, 60°, and 90°. This may be attributed to the initial deformation induced by gravity when the prototype is mounted onto the clamper.

**Figure 9:**
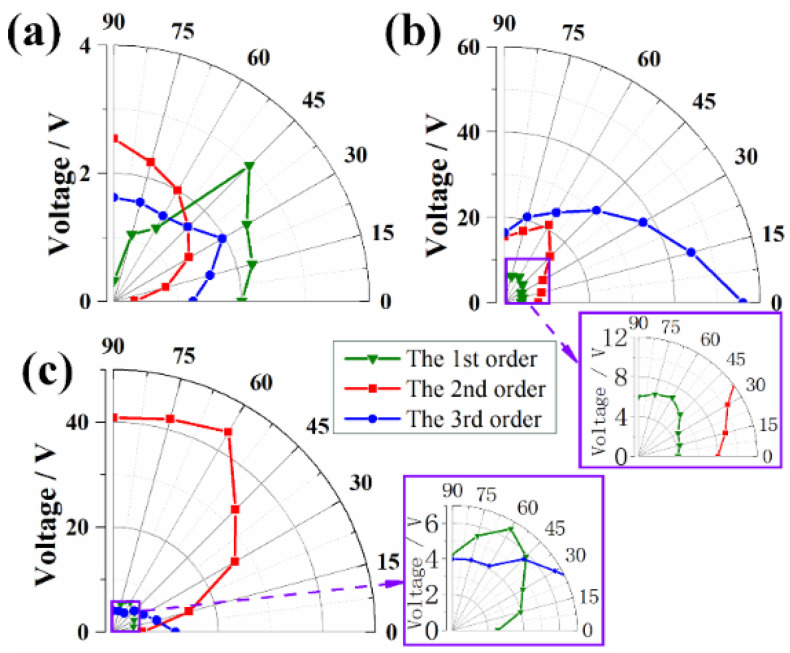
Output voltage of different beams versus α at the first three resonant frequencies: (**a**) upper beam, (**b**) horizontal beam, and (**c**) lower beam.

To further investigate the effect of α, the output voltage of different beams at the three resonant peaks, namely 2.6 Hz, 9.1 Hz, and 11.4 Hz, are concluded in Figure 9. When α = 0°, the horizontal beam exhibits a maximum voltage of 56 V at the third resonant peak, corresponding to the mode result in FEA. As α increases, the excitation direction deviates further from the direction of third-order mode, causing a continuous decrease to the voltage at the third-order resonant frequency. For the voltages at first- and second-order modes, the voltage of lower beam increases at the beginning (α = 0°~60°), and then maintains at a relative stable value at larger angles (α > 60°). This phenomenon may be attributed to the interdependence between the deformations of horizontal beam and upper and lower beams, whose vibration modes are the dominators at the first two resonant frequencies. For the upper beam, the largest voltage appears at the second frequency, whose bending mode is gradually triggered when α increases beyond 0°. The bending mode of upper beam cannot be activated by the vertical vibrations, and the small output voltages are mainly induced by the interdependence between different beams.

**Figure 10:**
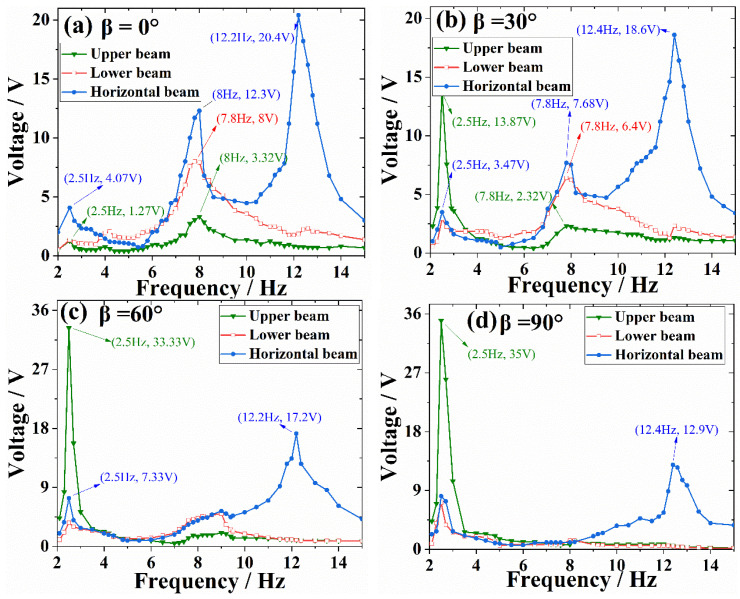
Open-circuit voltage versus frequency under horizontal excitation with different β.

**Figure 11:**
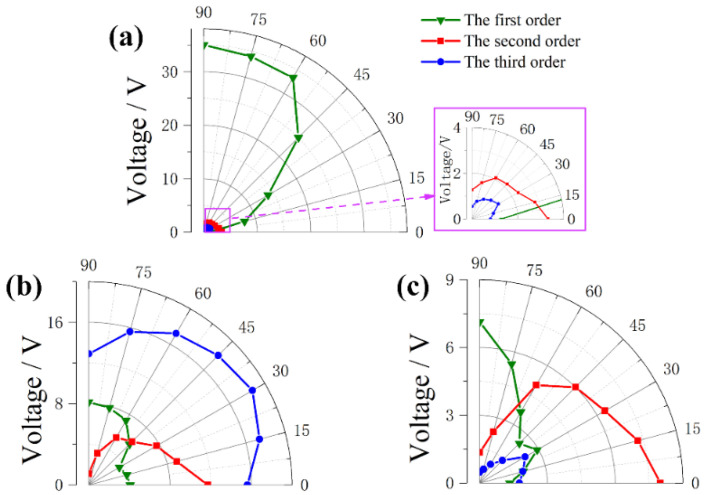
Output voltage of different beams versus β at the first three resonant frequencies: (**a**) upper beam, (**b**) horizontal beam, and (**c**) lower beam.

Figure 10 and Figure 11 indicate the voltage results under the horizontal vibrations. The bending mode of upper beam is activated at the frequency of 2.5 Hz when β increases beyond 0°, so the voltage produced by upper beam become larger and larger, reaching its largest value of 35 V when β = 90°. Meanwhile, the horizontal vibrations mainly triggered the higher modes that same with a conventional U-shaped beam. Therefore, the voltages of lower and horizontal beams are large when β = 0°, and gradually decreases with the increase of β.

**Figure 12:**
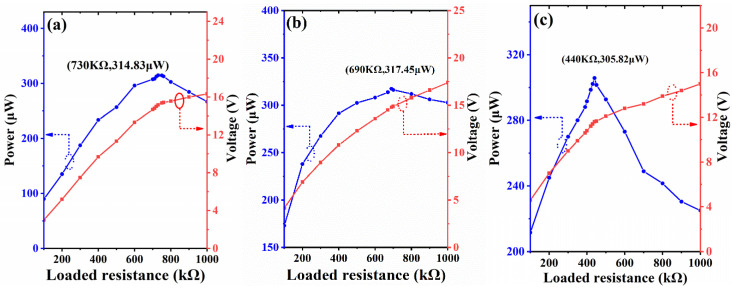
Output power versus loaded resistance under the maximum voltage for (**a**) upper beam, (**b**) lower beam and (**c**) horizontal beam.

The optimal impedance and maximum output power of proposed prototype are also tested. Firstly, the power is evaluated at the situation of highest output voltage of each beam. The two piezoelectric patches on upper beams are connected in parallel and stimulated by a 0.5 g, 2.5 Hz horizontal acceleration with β = 90°. Figure 12a shows its output voltage and power under varying load resistance. The optimal load resistance is 730 kΩ and with a maximum power of 314.83 μW. The lower beams are evaluated under the condition of 0.5 g, 9.1 Hz vertical vibration, parallelly connected patches and α = 90°. As shown in Figure 12b, the obtained optimal load resistance and maximum power are 690 kΩ and 317.45 μW. The horizontal beam is tested under the condition of 0.5 g, 11.4 Hz vertical vibration, parallelly connected patches and α = 0°. Figure 12c illustrates that the optimal load and output power are 440 kΩ and 305.82 μW. Then, the capacity of harvesting multidirectional vibration are also verified by loading a 0.5 g vertical excitation with α = 60° at the second-order resonance frequency. The results for upper, lower and horizontal beams are (690 kΩ, 7.02 μW), (690 kΩ, 230.09 μW), (690 kΩ, 112.23 μW), respectively. These experimental results indicate that the newly proposed U-shaped structure has good performances in harvesting multidirectional vibrations.

### 4.2. Discussions

The resonant frequency is a very important parameter for PVEHs. Herein, a simple comparison is conducted between the frequencies obtained from analysis and experiments. The results are shown in Table 2. It can be seen that the frequencies from FEA is a little higher than the measured ones. This circumstance can be attributed to the mechanical and electrical damping in the experimental prototype. Similarly, the frequencies from formulas are much higher than the experimental results (about 40% higher), due to the absence of proper damping ratio. It is a difficult work to model the mechanical and electrical damping with classical theories, and the obtained formulas may become too complex to guide the structural design. Therefore, the work focuses on the undamped condition to link the structural parameters and resonant frequencies. This simplification will inevitably enlarge the frequency values. Meantime, the connecting wires, adhesives for fixing PZT plates and proof mass will increase the equivalent mass of whole prototype, which can decrease the resonant frequencies and further expand the deviation.

Table 3 summarizes and compares some previously reported PVEHs with our proposed device in terms of normalized power density. It can be seen that the performance of the proposed PVEH has favorable comprehensive performance in frequency, multi-model, power and normalized volumetric power density (NVPD). Meanwhile, compared with the proposed PVEH, most 3D PVEHs had difficulty in achieving multiple resonant peaks in a certain frequency for every direction. Additionally, the proposed PVEH shows good performance in terms of NVPD compared with the existing multidirectional PVEHs, and the value can reach 0.1115 µW/(mm^3^g^2^Hz), which is close to many 1D PVEHs. Therefore, the proposed PVEH using improved U-shaped structural as a multidimension, multimodal energy harvesting architecture shows potential for practical applications in the daily environment.

Another critical issue in developing PVEH is the device reliability. Firstly, the structural dimension should be carefully determined to simultaneously guarantee the harvesting performances and keep the working stress within the allowable range. With the help of FEA software, the stress in brass substrate and piezoelectric plates are all evaluated. Except the stress concentration in some boundaries, the simulated stress is at the level of 50–100 MPa, lower than the extension strength of materials. Hundreds of thousands of acceleration cycles have been loaded to the PVEH prototype during characterization, and no significant attenuation happens in the output voltage. Secondly, the wires for power transmission are all simply suspended in air without additional protection, which might be a hidden trouble in device reliability. A sudden drag may break the conducting path and make the device out of work. Therefore, additional length of wire is reserved in the prototype to provide enough room for motion and drags. This situation is common for many reported experimental prototypes, and may be improved by using distribution frame and standard connectors.

### 4.3. Applications

**Figure 13:**
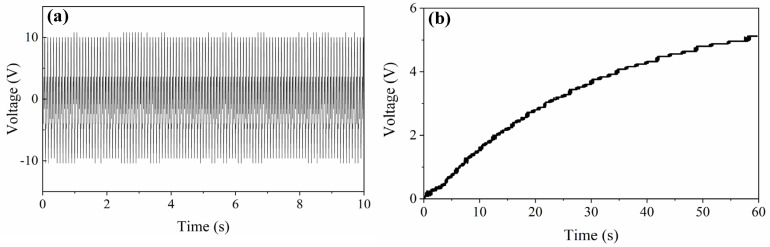
Applying proposed PVEH in harvesting sustained vibration. (**a**) Output voltage of proposed PVEH; (**b**) The voltage over charged capacitor.

First, the proposed PVEH is triggered by shaker, and the vibration parameters are set according to the vibration in instrument panel of a vehicle [37]. Figure 13 indicates the open-circuit voltage of horizontal beam. Under the sustained vibration, the proposed PVEH can generate a steady voltage about 10 V and charge a 47 μF capacitor to 5 V in one minute with the help of rectifier (LTC3588-1).

**Figure 14:**
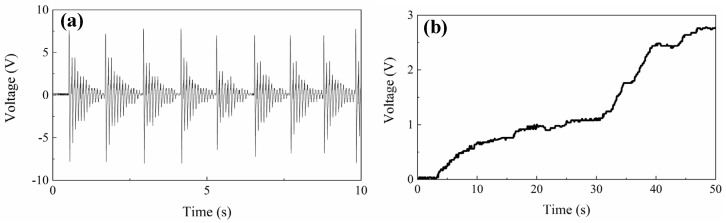
Applying proposed PVEH in harvesting stamping vibration. (**a**) Output voltage of proposed PVEH; (**b**) The voltage over charged capacitor.

Then, the biomechanical energy in human walking is harvested by mounting the PVEH at waist. Stamping the feet gently in a frequency about 1 Hz will load a sustained shock stimulation to PVEH. The captured voltage is shown in Figure 14a, in which a decaying wave signal by attenuating free vibration with a resonant frequency of 10.8 Hz is obtained due to electromechanical coupling and mechanical damping. As shown in Figure 14b, a 22 μF capacitor can be charged to 2.8 V after 80 steps by slow stamping (0–30 s) or fast stamping (30–60 s).

**Figure 15:**
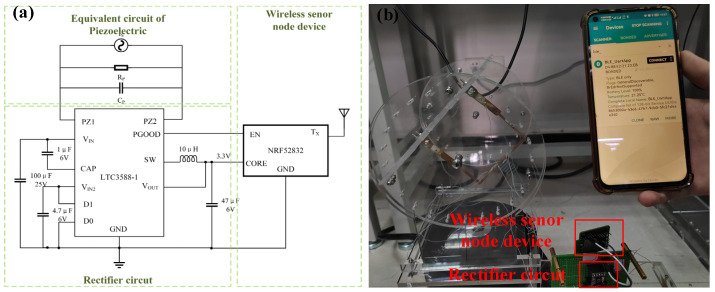
Applying proposed PVEH in powering a wireless sensor node. (**a**) Circuit diagram of the wireless sensor node device; (**b**) Picture of signal transmission through Bluetooth.

Last, a wireless sensor node (NRF 52832) based on Bluetooth is connected to the output port of a harvesting circuit (Figure 15), which manages and stores the output voltage of proposed PVEH. The device is sustainably stimulated by the former mimetic vibration, and the proposed system enables to drive the sensor node and transmit the temperature signal to a cellphone. Considering the real-time output ability of proposed PVEH, the powered wireless sensor node can work well in the situations without continuous task execution, such as monitoring the environmental temperature/humidity. Meanwhile, with the help of energy storage devices (e.g., the abovementioned capacitors), the node can still get the required power when there are no vibrations.

## 5. Conclusions

This paper proposed a U-shaped structure with cross-connected beams for harvesting energy from multidirectional vibrations in daily environment. The multidirectional and multimodal ability of proposed PVEH originates from the combination of bi-directional feature of conventional U-shaped beam and the additional bending direction of cross-connected beams. A finite element simulation reveals the potential advantages of improved structure in low resonant frequency and good responses to vibrations in different directions. An experimental prototype is tested by a shaker system with the help of tailored clampers. The results show that the proposed PVEH can continuously produce favorable electric power when the vibrations are vertically or horizontally loaded with different orientation angles. Moreover, three resonant frequencies are produced in the range of 2–15 Hz, giving the PVEH prototype a good adaptation to the frequency spectrum of daily vibrations. Capacitors can be charged by the energies converted from mechanical vibrations and human motions. The stored energy can be used to power a Bluetooth-based wireless sensor node device. These results reveal that the proposed harvester features multidirectional and multimodal properties for the daily vibrations. Moreover, the insufficient theorical modelling needs further investigation in future work, especially on the electromechanical coupling analysis.

## Figures and Tables

**Table 1:** The dimensions of components in U-shaped structure (Unit: mm).

Components	Valid Length	Width	Thickness
PZT-5A	12	8	0.2
Horizontal beam	100	10	0.2
Lower beam	50	10	0.2
Upper beam	40	10	0.2

**Table 2:** Comparison of resonant frequencies from simulation and experiment (Unit: Hz).

Orders	Measured	Simulated	Deviation
1st	2.6	2.26	0.34
2nd	9.1	10.79	1.69
3rd	11.4	13.89	2.35

**Table 3:** Comparison of the proposed and some existing PVEHs.

Works	Excitations (1D/2D/3D)	Acc. (g)	Fre. (Hz)	Power (µW)	Volu. (mm^3^)	NVPD (µW/(mm^3^g^2^Hz))
[34]	1D	0.041	27.5	93	6300	0.3139
[35]	1D	1	160	2490	880	0.0177
[23]	1D	0.1	12	442	8400	0.4383
[36]	2D	3	18	963.9	3120	0.00191
[32]	3D	0.5	23.7	9.2	4256	0.00036
[30]	3D	0.008	4.56	-	982	-
[28]	3D	0.5	8	110.3	3480	0.0158
[31]	3D	1	2.9	306	4507	0.0234
This work	3D	0.5	2.5	314	4586	0.1115

## Data Availability

The data presented in this study are available upon request from the corresponding author. The data are not publicly available due to the intellectual property.
